# Lack of formal regulatory definitions of off-label medication use in children: an analysis using agency inquiry and text mining

**DOI:** 10.3389/fphar.2025.1750718

**Published:** 2026-01-09

**Authors:** Christina Gade, Ulrik Lausten-Thomsen

**Affiliations:** 1 Department of Clinical Pharmacology, Copenhagen University Hospital – Bispebjerg and Frederiksberg, Copenhagen, Denmark; 2 Department of Clinical Medicine, University of Copenhagen, Copenhagen, Denmark; 3 Department of Neonatology, Copenhagen University Hospital – Rigshospitalet, Copenhagen, Denmark; 4 Department for Congenital Disorders, Statens Serum Institut, Copenhagen, Denmark

**Keywords:** definition, off-label, pediatric pharmacology, pharmacology, regulatory agencies

## Abstract

**Objectives:**

This study investigates the formal definitions of “off-label” medication use among five major Western regulatory authorities—FDA, EMA, Health Canada, MHRA, and TGA. The primary research question is whether these agencies provide explicit, official definitions of off-label use.

**Design:**

The study employs a mixed-methods design, combining direct inquiries via standardized questionnaires with AI-assisted text mining of publicly available regulatory documents.

**Setting:**

Regulatory agencies at a national level across North America, Europe, and Oceania.

**Participants:**

Five agencies, with data collected through direct contact and automated document analysis; no human participants were involved.

**Intervention:**

Analysing agency webpages and documents for sentences that resemble formal definitions, followed by manual review and categorization based on linguistic and contextual criteria.

**Main outcome:**

The presence or absence of official definitions, the content and clarity of any definitional statements, and their prominence within regulatory documents.

**Result:**

None of the agencies provide a formal, official definition of off-label use. However, all agencies’ publicly available documents contain statements that resemble definitions, generally describing off-label use as prescribing beyond the conditions approved by the drug’s marketing authorization. Despite similarities in language, the clarity and prominence of these statements vary across agencies.

**Conclusion:**

The lack of formal off-label definitions may contribute to legal ambiguity, clinical uncertainty, and challenges in guideline development, particularly affecting paediatric populations where off-label prescribing is common. The regulatory agencies should adopt clear, standardized official definitions of off-label use to improve transparency, legal coherence, and patient safety.

## 1 Introduction

Appropriate medications are often unavailable for special populations, which forces healthcare providers to prescribe and administer medications outside the conditions approved by regulatory authorities. This practice is especially common in paediatric populations, where the limited availability of age-appropriate and approved medicines inevitably results in compromised medication efficacy and safety from time to time (Paediatric Regulation | European Medicines Agency (EMA), 2015; Mørk et al., 2022).

In 2007, the Paediatric Regulation was introduced with the objective of improving the availability of authorized medicines for children, ensuring better access to age-appropriate treatments, and promoting the development of paediatric-specific medicines to enhance the safety and efficacy of treatments for the paediatric population (Paediatric Regulation | European Medicines Agency (EMA), 2015). However, anticipating that this Paediatric Regulation would immediately and completely resolve this issue proved to be overly optimistic. It is a reality that certain populations and conditions are likely to remain underserved, either due to pharmaceutical companies’ reluctance or inability to pursue regulatory approval for these cases (Mørk et al., 2022; Ward et al., 2018). A systematic review found substantial variability in, knowledge, perceptions of off-label use, and particularly awareness of off-label prescribing in children (Balan et al., 2015) and establishing a standardized definition is a first essential step for improving medication safety and efficacy for the paediatric population.

Therefore, we must confront this limitation within real-life clinical practice. A good starting point is to reach a consensus on how this practice is defined. In 2008, a Delphi survey conducted among European experts sought to address the lack of common definitions for off-label medicine use by developing consensus-driven definitions for “off-label” and “unlicensed” terms to be used for research and regulatory purposes (Neubert et al., 2008). This survey revealed substantial variations in the interpretation of off-label and unlicensed use, which led to proposed standardized definitions. These definitions, agreed upon by the majority of experts, distinguish between off-label use—where an authorized drug is used in ways not approved by its marketing authorization—and unlicensed use, which refers to the use of drugs that have never received European Marketing Authorization. The Delphi panel encouraged that these definitions should be circulated within the scientific community and recommended to be adopted by relevant regulatory authorities.

We here aim to consult the major Western regulatory authorities to determine whether they officially define off-label medicine use in the paediatric population, with the goal of exploring whether the consensus recommendations on off-label medicine use have been adopted.

## 2 Methodology

We employed a mixed-method approach combining direct correspondence and AI-assisted text mining.

### 2.1 Regulatory Survey via direct communication

We contacted five key regulatory agencies—U.S. Food and Drug Administration (FDA), European Medicines Agency (EMA), Health Canada, the United Kingdom’s Medicines and Healthcare products Regulatory Agency (MHRA), and Australia’s Therapeutic Goods Administration (TGA)—via email between 1st December 2024 and 1st March 2025. A standardized questionnaire was used to ensure consistency in the information requested, focusing on official definitions, classification criteria, and relevant regulatory frameworks for off-label use (see Supplementary Appendix). If no response was received within 4 weeks, a follow-up request was sent. All responses were recorded, categorized, and compared to identify commonalities and divergences across agencies.

### 2.2 AI-assisted text mining of regulatory websites

For all agencies, we systematically analysed their official websites and documents. We developed a multi-stage text mining pipeline using Python (v3.11) to retrieve, parse, and analyse relevant content (Python Software Foundation, 2025). A custom web crawler collected HTML and PDF documents from each agency’s domain, limiting depth to two link levels and avoiding external or redundant content. Text was extracted, segmented into sentences (30–300 characters), and filtered for relevance.

Natural language processing (NLP) algorithms have previously been described useful in analyzing large amount of pharmacological relevant text from government agencies (Bergman et al., 2023), and here we used a pre-trained Sentence-BERT (SBERT) model (Reimers and Gurevych, 2019), to generated semantic embeddings for both the corpus and definition-related query phrases (e.g., “definition of off-label use”). These embeddings enabled semantic similarity searches to identify candidate sentences.

### 2.3 Definition-likeness and prominence scoring

Candidate sentences were manually reviewed and categorized as definitional, contextual, or irrelevant, and the top-3 was selected. Each candidate sentence was subsequently scored for “definition-likeness” on a 5-point scale based on six linguistic heuristics: (1) term-first structure; (2) definitional cue phrases; (3) presence of a linking verb; (4) semantic clarity; (5) self-contained phrasing; and (6) absence of narrative or example-based content, following established criteria (Navigli et al., 2010). See Supplementary Table S1 for heuristic scoring framework.

Separately, a 5-point “prominence score” was assigned based on webpage positioning and emphasis: (1) HTML tags (e.g., headings, glossary entries); (2) location in the document (e.g., top 25%); (3) contextual framing (e.g., within dedicated definition sections); and (4) formatting (e.g., bold or isolated text). All scoring was conducted independently by two reviewers, with discrepancies resolved by consensus. See Supplementary Table S2 for scoring framework.

## 3 Results

All responses to direct written inquiries to the five major Western agencies are summarized in Table 1. Four of five agencies responded as having no official definition of the concept ‘off-label medication’, whereas the fifth agency, the Healthcare products Regulatory Agency (MHRA) in the United Kingdom, did not specify whether they endorsed an official definition, but referred to separate documents on their webpage.

**TABLE 1:** Regulatory agencies answer to the inquiry.

Agency	Response	Official definition	DL-score
FDA	“FDA does not have an official definition of ‘off-label’ or ‘off-licence’ use of a medication”	No	0
EMA	“We have not defined off-label use that you could present as an ‘EMA definition’”	No	0
Health Canada	“Health Canada does not have an official definition of the term.”	No	0
MHRA	“In the first instance we recommend you review the information we have published on our website on this topic, which can be accessed from the two links below: https://www.gov.uk/drug-safety-update/off-label-or-unlicensed-use-of-medicines-prescribers-responsibilities https://assets.publishing.service.gov.uk/media/645e19f5ad8a03000c38b3bc/The_supply_of_unlicensed_medicinal_products__special_GN14.pdf	No	0
TGA	“The therapeutic goods Act 1989 (the Act) does not regulate clinical practice. ‘Off-label use’ is a clinical decision, made at the discretion of the treating clinician. This clinician is responsible for obtaining informed consent from their patient (which includes telling them if the use is off-label) and ensuring that the medicine or device selected is the most appropriate treatment option.”	No	0

DL-score, definition-like score (see text for details).

The AI-assisted pipeline effectively retrieved and analysed definition-like sentences related to off-label medication use from the official websites of five major regulatory agencies. Notably, each agency’s web presence included at least one sentence that received a maximum Definition-Likeness Score (DL-score) of 5, indicating a prototypical definitional construction. See Table 2 for details.

**TABLE 2:** Definition-like sentences from official webpages.

Agency	Definition-like sentence	DL-score	WP-score	Link
FDA	“Unapproved use of an approved drug is often called “off-label” use.”	5	5	https://www.fda.gov/patients/learn-about-expanded-access-and-other-treatment-options/understanding-unapproved-use-approved-drugs-label?
	“…Prescribe or administer the drug in clinical practice for an unapproved use not described in the approved labeling (i.e., ‘off-label')”	3	3	https://www.fda.gov/consumers/consumer-updates/understanding-regulatory-terminology-potential-preventative-and-therapeutic-drugs-covid-19
	“Treat with medications based on adult studies with limited or anecdotal pediatric experience (off-label use)”	2	2	https://www.fda.gov/files/drugs/published/Pediatric-Drug-Development--Regulatory-Expectations.pdf
EMA	“Use of a medicine for an unapproved indication or in an unapproved age group, dosage, or route of administration”	5	5	https://www.ema.europa.eu/en/glossary-terms/label-use
	“Situations where a medicinal product is intentionally used for a medical purpose not in accordance with the terms of the marketing authorization”	5	3	www.ema.europa.eu/en/documents/scientific-guideline/guideline-good-pharmacovigilance-practices-annex-i-definitions-rev-5_en.pdf
	“Off-label use (e.g., not authorised for one or more paediatric age groups)”	2	2	www.ema.europa.eu/en/documents/scientific-guideline/overview-comments-received-reflection-paper-use-extrapolation-development-medicines-paediatrics-ema1897242018-revision-1_en.pdf
Health Canada	“Off-label use refers to any intentional use of a drug as prescribed by a qualified healthcare professional, but that is not covered by the terms of its marketing authorization”	5	3	www.canada.ca/content/dam/hc-sc/documents/services/drugs-health-products/medeffect-canada/adverse-reaction-reporting/drugs-devices/guidance/Mandatory-reporting-hospitals-eng.pdf
	“Off-label use of a marketed drug (i.e., a use not in accordance with the labelled uses or conditions of use for which the drug has received market authorization)	3	4	www.canada.ca/en/health-canada/services/drugs-health-products/drug-products/announcements/notice-statement-investigational-use-marketed-drugs-clinical-trials.html
	“…many medications that are prescribed for children and youth are prescribed off-label, which means outside of the approved use of the drug in Canada”	3	3	www.canada.ca/en/health-canada/services/drugs-health-products/drug-products/pediatrics.html
MHRA	“the use of unlicensed medicines or use of medicines outside the terms of the licence (i.e., ‘off-label')”	3	3	www.gov.uk/drug-safety-update/off-label-or-unlicensed-use-of-medicines-prescribers-responsibilities
	“Off-label’ prescribing means the person prescribing the medicine is using it for a different purpose or at a different dose to that specified in the licence”	5	3	www.gov.uk/government/publications/chm-report-into-the-safety-implications-of-proposed-puberty-blockers-legislation-factsheet/commission-on-human-medicines-report-into-the-safety-implications-of-proposed-puberty-blockers-legislation-factsheet
	“…Offer a vaccine that is ‘off-label’. This means that, although the vaccine is authorised for use, it’s being used in a way that is slightly different from the strict terms laid down in its license … ”	3	4	www.gov.uk/government/publications/off-label-vaccine-leaflets/off-label-vaccines-an-introductory-guide-for-healthcare-professionals-text-version
TGA	“Medicines are often used in clinical practice for conditions other than those approved by the TGA. This practice is known as ‘off-label’ use.”	4	4	www.tga.gov.au/resources/publication/publications/medicines-repurposing-program
	“Off-label use’ is using a product for a reason not listed as one of the indications for use in the Australian register of therapeutic goods (ARTG)”	5	5	www.tga.gov.au/resources/guidance/understanding-regulation-label-use-medical-devices
	“Off-label’ use is, by definition, outside the terms of the marketing authorisation and therefore difficult to regulate. This applies to paediatric use whether it is on- or off-label”	5	4	www.tga.gov.au/sites/default/files/2024-06/guideline-conduct-pharmacovigilance-medicines-used-by-paediatric-population-ema.pdf

DL-score, definition-like score; WP, score, webpage-prominence score.

Across the five regulatory agencies examined, there was a notable convergence in the core semantic content of definition-like sentences describing off-label use. Most definitions referenced the use of a medication “outside the terms” of its approved marketing authorization or product label, reflecting a shared regulatory understanding of off-label use as a deviation from formal approval conditions. Additionally, common linguistic features such as cue phrases (“refers to”, “means”, “is defined as”) and term-first constructions were recurrent among high-scoring definitions, suggesting alignment in discursive framing.

Despite this common core, there were discernible differences in specificity, target populations, and scope. For example, Health Canada and the TGA explicitly included veterinary or paediatric contexts in several of their definitions, extending the scope beyond human adult medicine. The EMA’s definition emphasized routes of administration and dosage forms, reflecting a more technical nuance, while the MHRA often embedded off-label use within broader discussions of unlicensed medicines, potentially blurring distinctions in regulatory terminology.

Furthermore, the placement and formality of these definitions varied: some were embedded in glossaries or standalone sections (e.g., EMA, TGA), lending them higher institutional authority, while others appeared in narrative contexts or PDF annexes, which could dilute their perceived normative weight. Indeed, all regulatory agencies provided definition-like statements in various locations on their official websites.

The European Medicines Agency (EMA) stood out in particular: it featured a sentence with a maximal Definition-Likeness Score (5/5), exhibiting a structure highly characteristic of formal definitional language. This sentence was furthermore located within the agency’s online glossary (https://www.ema.europa.eu/en/glossary-terms/label-use) and its placement in a dedicated definitional context rendered it virtually indistinguishable from an official EMA-sanctioned definition. The positioning not only enhanced accessibility for users but also conferred a degree of institutional authority to the statements, thereby reinforcing their interpretive weight.

## 4 Discussion

None of the five major Western regulatory authorities revealed official definitions of off-label medicine use when contacted directly via email, which clearly indicates that they have chosen not to adopt the Delphi expert-written definition of off-label use for regulatory purposes. While the United Kingdom did not confirm having a definition, it did not provide a clear statement either (see Table 1). However, a review of the materials available on the official websites of all regulatory bodies revealed language that closely resembled the definition recommended by the Delphi expert panel—namely, that off-label use is “where an authorized drug is used in ways not approved by its marketing authorization” (see Table 2).

These references often echoed definitions found in scientific literature, with the most explicit descriptions frequently appearing in patient-oriented materials. It is important to note that scientific literature typically offers clearer and more operational definitions aimed at supporting research, clinical decision-making, and reproducibility. In contrast, regulatory language tends to be broader and more flexible, accommodating diverse legal and political contexts. This pragmatic approach can lead to imprecise terminology, which poses challenges for clinical practice, legal interpretation, and policy formulation (McHughen, 2016).

This study has several strengths, including the use of a mixed-method approach combining direct correspondence with regulatory agencies and advanced AI-assisted text mining to systematically analyse official web content. However, there are also limitations to consider. The reliance on publicly available online content may not fully capture internal or unpublished regulatory policies. Also, generally the use of AI-assistance in text mining may be subjective to computational errors and variations depending on the choice of natural language processing (NLP) algorithm (Stammers et al., 2025). Finally, the interpretation of definitional language by the authors can be somewhat subjective, despite the structured scoring system, which may influence the consistency of the analysis.

The approach employed in this study differs from other research efforts that have sought to establish a consensus definition or develop off-label frameworks. Most of these previous initiatives have been conducted externally or through consultations with only a single regulatory agency. In contrast, the present study distinguishes itself by collecting official or semi-official statements from all major Western regulatory agencies. This methodology emphasizes examining existing definitions—or the absence thereof—rather than attempting to formulate a new, overarching definition.

The lack of a unified regulatory definition contributes to uncertainty among healthcare professionals, hampers the development of consistent clinical guidelines, and limits the comparability of research across jurisdictions (Reimers and Gurevych, 2019). In paediatric pharmacology—where off-label prescribing in regard to indication, dose, route, formulation, ect. Is particularly common—this ambiguity complicates pharmacovigilance efforts, hampers ethical risk communication, and reduces transparency in treatment decisions.

Therefore, we urge regulatory authorities to issue a formal, unambiguous definition of off-label medicine use. Such a definition should reflect prevailing implicit practices and be aligned with terminology already present in public-facing materials. Codifying this definition would enhance legal clarity, support the development of clinical guidelines, and ultimately improve patient safety and clinical governance.

## 5 Conclusion

Our analysis reveals that major Western regulatory authorities do not formally define off-label medicine use, despite employing language that closely mirrors expert consensus definitions. This absence of a clear, unified definition creates ambiguity for clinicians, researchers, and policymakers—particularly in fields like paediatric pharmacology, where off-label use is prevalent. We therefore recommend that regulatory bodies adopt an explicit, standardized definition aligned with existing public-facing terminology to promote legal clarity, improve clinical guidance, and support patient safety.

## Data Availability

The original contributions presented in the study are included in the article/Supplementary Material, further inquiries can be directed to the corresponding author.
