# Supplementary material for: Lack of formal regulatory definitions of off-label medication use in children: an analysis using agency inquiry and text mining
Source: Front Pharmacol. 2026 Jan 9;16:1750718. doi: 10.3389/fphar.2025.1750718 (PMC12827503; doi:10.3389/fphar.2025.1750718)
Supplement: Supplementary file 1 [file Supplementaryfile1.docx]

**Appendix**

**Supplement 1:** the letter sent to the Regulatory Agencies

To Whom It May Concern,

**Concerning definition of "off-label" medication use**

We are currently conducting a scientific study focused on the definitions of "off-label" and "off-license" medication use (or similar terminology), with a particular emphasis on pediatric applications. This scientific and regulatory initiative has been launched by researchers from The Danish Center for Pediatric Clinical Pharmacology, Copenhagen University Hospital, and Copenhagen University.

In this regard, we would appreciate your assistance in answering the following questions:

1. 1) Does the [*name of the agency*] have an official definition of "off-label use of medication"? If so, how do you define it?
2. 2) Does the [*name of the agency*] have an official and separate definition of "off-label use of medication" for the pediatric populations ? If so, how do you define it?
3. 3) Does the [*name of the agency*] differ between “off-label” and “off-licence”? If so, how do you define “off-licence”?
4. 4) Could you please direct us to any references, official reports, directories on your website, or similar resources that provide these definitions in writing?

Thank you for your attention to this inquiry. We look forward to your response.

On behalf of the author group,

**Supplementary table 1.**

| **Table S1: Definition-Likeness Score Interpretation Table** | | |
| --- | --- | --- |
| Score | Label | Interpretation |
| 1 | Not definition-like | Sentence is vague, narrative, or unrelated; no identifiable definition structure. |
| 2 | Weakly definition-like | Sentence has minimal cues (e.g., term or example), but lacks structure or clarity. |
| 3 | Moderately definition-like | Partial definition: some structural features (e.g., copula or cue), but not complete or precise. |
| 4 | Clearly definition-like | Sentence functions well as a definition but may lack one feature (e.g., structure or self-containment). |
| 5 | Prototypical definition | Complete, clear, stand-alone definition using formal structure and precise language. |

**Supplementary table 2.**

| **Table S2: Prominence Score Interpretation Table** | | |
| --- | --- | --- |
| Score | Label | Interpretation |
| 0 | Not prominent | Buried in footnotes, appendices, or deeply nested lists; unlikely to be noticed. |
| 1 | Low prominence | Appears in an example or as a parenthetical mention without clear focus. |
| 2 | Slightly prominent | Embedded mid-document in a paragraph; no visual or structural emphasis. |
| 3 | Moderately prominent | Within a relevant section but not highlighted (e.g., mid-page body text). |
| 4 | Highly prominent | Near the top of a section, under a heading, or clearly marked as a definition. |
| 5 | Very highly prominent | Positioned at the top of the page, in a glossary, heading, or summary block. |
